# Editorial: Long Non-coding RNAs and Immunity

**DOI:** 10.3389/fimmu.2019.02378

**Published:** 2019-10-09

**Authors:** Grace J. Kwon, Jorge Henao-Mejia, Adam Williams

**Affiliations:** ^1^Department of Genetics and Genome Sciences, University of Connecticut Health Center, Farmington, CT, United States; ^2^The Jackson Laboratory for Genomic Medicine, Farmington, CT, United States; ^3^Department of Pathology and Laboratory Medicine, University of Pennsylvania, Philadelphia, PA, United States; ^4^Institute for Immunology, Perelman School of Medicine, University of Pennsylvania, Philadelphia, PA, United States; ^5^Division of Transplant Immunology, Department of Pathology and Laboratory Medicine, Children's Hospital of Philadelphia, University of Pennsylvania, Philadelphia, PA, United States

**Keywords:** long non-coding RNA, immunity, circular RNA, X-chromosome inactivation, viral infection, rickettsial infection, GWAS

Next-generation sequencing has shown that the majority of the human genome does not encode proteins, yet many such “non-coding” regions are actively transcribed into RNA. In particular, long non-coding RNAs (lncRNAs) have received considerable interest for their essential roles in numerous biological processes. Defined as non-protein-coding transcripts longer than 200 nucleotides, lncRNAs are frequently expressed in a tissue-specific or developmental stage-specific manner (1, 2). Multiple studies have shown lncRNAs to be dysregulated in disease, and genome-wide association studies (GWAS) have also identified numerous single nucleotide polymorphisms (SNPs) within lncRNAs (3–5). These features distinguish them as attractive candidates for therapeutic targets or biomarkers. While still in its infancy, the dissection of lncRNA biology has unveiled critical roles in immune cell development and function. However, as the functions of most lncRNAs are yet to be determined, the field must fill a considerable void to understand the breadth of their roles in immunity. This Research Topic focuses on how lncRNAs contribute to immune function in a variety of different contexts, and what key questions drive this rapidly expanding field.

## Dosage Compensation and X-linked Immune Genes

Females have a more responsive immune system than males (6, 7), potentially driving a higher predisposition to autoimmune diseases such as systemic lupus erythematosus (SLE) (8). The X chromosome carries a significant number of immune-related genes, but how this translates to sex disparities in immune diseases remains to be determined. *Xist* is one of the best characterized lncRNAs, and functions as an effector of dosage compensation in female cells. *Xist* maintains inactivation of one X chromosome by binding along its length and recruiting repressive factors to silence transcription. Immune cells, however, regulate X chromosome inactivation through diverse mechanisms that are not limited to the use of *Xist* RNA, as recently revealed by Syrett et al. For instance, the inactivated X in female murine plasmacytoid dendritic cells (pDCs) lacks both *Xist* RNA and the repressive chromatin modification H3K27me3. Interestingly, pDCs from female mice that spontaneously develop SLE-like disease show increased biallelic expression of the X-linked gene *Tlr7*, as compared to healthy female mice. Further delineating the mechanisms of X chromosome inactivation in immune cells will be necessary before we can develop a complete understanding of how sex and sex-linked genes contribute to autoimmune diseases.

## lncRNAs in Rickettsial Infection

The *Rickettsia* genus of Gram-negative obligate intracellular bacteria is transmitted to humans via arthropod vectors, including ticks, fleas, and lice, and can infect the vascular endothelium to cause illnesses such as Rocky Mountain spotted fever. There are increasing concerns over the prevalence of *Rickettsia* due to climate change and the spread of vector populations. Understanding rickettsial infection and developing novel therapeutics to mitigate its spread through the vascular endothelium will thus be crucial in the near future. Chowdhury et al. have begun to address the roles of lncRNAs in the host response to *Rickettsia* species using a mouse model of *R. conorii* infection. The authors identified two enhancer lncRNAs affecting expression of nearby genes, *Id2* and *Apol10b*, which have not been previously studied in rickettsial infection. Furthermore, these two lncRNAs exhibit differential expression in infected macrophages compared to endothelial cells, emphasizing the cell type-specificity of lncRNAs. The resulting genome-wide analysis of lncRNA expression during rickettsial infection establishes a foundation to further study the host immune response to *Rickettsia* species and reveals cell type-specific signaling pathways mediated by enhancer lncRNAs.

## Viral and Host lncRNAs in Antiviral Immunity

Some of the most interesting advancements in our understanding of lncRNAs in immunity come from the study of their roles in viral infection. While it would be intuitive to assume host lncRNAs exist to suppress viral infections, it has been shown that host lncRNAs can actually increase viral replication and pathogenesis during certain infections (9). This begets the need to further examine what purpose both host and viral lncRNAs serve, and how it may change in the context of non-infected and infected states. Wang reviews in detail the known lncRNAs involved in multiple steps of the viral life cycle and infection.

## Lncing GWAS SNPs to Disease Pathogenesis

GWAS have provided a more complete understanding of complex diseases. Of the more than 70,000 variant-trait associations cataloged from GWAS (10), a majority of these map to non-coding regions of the genome, including those containing lncRNAs. Despite this, the impact of only a few SNPs on lncRNA function has been described. A major limitation in the field is that the function of most lncRNAs is unknown. In addition, while SNPs in lncRNAs likely affect their secondary and tertiary structure, we cannot currently predict how this will alter their function. Castellanos-Rubio and Ghosh review the disease-associated lncRNA SNPs that have been described thus far. Further inquiry and development of tools to interpret lncRNA structure and function will be necessary before the impact of lncRNA SNPs (and other genetic variants) on disease can be fully realized.

## Circular RNAs as a New Class of Immune Regulators

While lncRNAs typically display features associated with linear messenger RNA, such as 5′ capping, alternative splicing, and polyadenylation, non-coding RNA loci can also be transcribed to form circular RNA (circRNAs). These circRNAs form closed loops without 5′ capping or polyadenylation and can contain multiple exons. Similar to lncRNAs, circRNAs are frequently expressed in a tissue-specific manner, undergo chemical modifications, and function through varied mechanisms. Yang et al. review the mechanisms of circRNA biogenesis, summarize the current understanding of their function in immunity, and present approaches and limitations to studying circRNA function.

## Conclusion

lncRNAs are now an established class of functional molecules with physiological relevance shown in nearly every human organ system. Their roles in immunity have been identified in immune cell differentiation, maintenance, and effector function. Given that only a fraction of lncRNAs have been functionally characterized a significant wealth of insight awaits discovery. Moreover, as personalized medicine begins to drive the use of genetic tools in the clinic, we can expect the identification of clinically-relevant lncRNA variants and the development of therapeutics that target lncRNA function.

## Author Contributions

AW and GK wrote and edited the manuscript. JH-M edited the manuscript.

### Conflict of Interest

The authors declare that the research was conducted in the absence of any commercial or financial relationships that could be construed as a potential conflict of interest.
